# 3D printing technology is a more accurate tool than an experienced surgeon in performing femoral bone tunnels in multi‐ligament knee injuries

**DOI:** 10.1002/jeo2.70159

**Published:** 2025-02-06

**Authors:** Núria Fernández‐Poch, Ferran Fillat‐Gomà, Mireia Gamundi, Giovanni Grillo, Christian Yela‐Verdú, Sergi Gil‐Gonzalez, Xavier Pelfort

**Affiliations:** ^1^ Orthopaedics Department, Parc Taulí Hospital Universitari, Institut d'Investigació i Innovació Parc Taulí (I3PT‐CERCA) Universitat Autònoma de Barcelona Sabadell Spain; ^2^ 3D Surgical Planning Lab, Parc Taulí Hospital Universitari, Institut d'Investigació i Innovació Parc Taulí (I3PT‐CERCA) Universitat Autònoma de Barcelona Sabadell Spain

**Keywords:** 3D printing, femoral tunnels, knee, multi‐ligament reconstruction, patient‐specific instrumentation

## Abstract

**Purpose:**

Current surgical methods for multi‐ligament knee reconstruction involve the creation of several reconstruction tunnels in the distal femur. However, the limited bone mass in the knee increases the risk of tunnel convergence. Increasing the accuracy of tunnel direction can minimize tunnel collision during anatomical reconstruction. 3D‐printed patient‐specific instrumentation (PSI) has gained prominence in orthopaedic surgery due to its precision. This study aims to compare the accuracy of PSI with that of the ‘freehand’ approach by an experienced surgeon for drilling the medial and lateral femoral tunnels while adhering to the recommended angulations for multi‐ligament knee injuries.

**Methods:**

Ten cadaveric knees underwent computerized tomography (CT) scans to identify anatomical femoral attachments of the lateral collateral ligament (LCL), popliteal tendon (PT), medial collateral ligament (MCL) and posterior oblique ligament (POL). Using Materialise Mimics Medical v25.0 software, virtual planning of a bone tunnel for each ligament was performed, and a total of four tunnels per knee were obtained. Ten PSIs were designed for five knees: five for the medial side and five for the lateral side. The first five knees were operated on via PSI, and the other five knees were operated on by an experienced surgeon using freehand drilling based on preoperative plans. The angular deviation and entry point were assessed by overlaying post‐operative CT images onto preoperative CT images.

**Results:**

In the freehand group, the median angular deviation was 22.3°, with an interquartile range (IQR) of 17.6–25.2°. The PSI group presented a significantly greater accuracy in angular deviation for femoral tunnels of 5.7°, with an IQR of 4–8.2° (*p* < 0.001). Compared with that in the preoperative planning group, the median entry point distance in the freehand group was 5.5 mm, with an IQR of 2.6–8.8 mm. The PSI group had a median entry point distance of 4.2 mm, with an IQR of 3.6–5.7 mm (*p* = n.s).

**Conclusions:**

Compared with the freehand technique performed by an experienced surgeon, PSI demonstrated significantly greater accuracy in terms of the mean angular deviation.

**Level of Evidence:**

Level V.

**Abbreviations**
ACL: anterior cruciate ligament
CT: computerized tomography
ICP: iterative closest point
IQR: interquartile range
LCL: lateral collateral ligament
MCL: medial collateral ligament
PCL: posterior cruciate ligament
PLA: polylactic acid
POL: posterior oblique ligament
PSI: patient‐specific instrumentation
PT: popliteal tendon
PVA: polyvinyl alcohol

## INTRODUCTION

A multi‐ligament knee injury occurs when two or more of the four major knee ligaments are injured, namely, the anterior cruciate ligament (ACL), posterior cruciate ligament (PCL), lateral collateral ligament (LCL) or posterolateral corner, and medial collateral ligament (MCL) or posteromedial corner [23]. Multi‐ligament knee injuries are rare and can have devastating consequences for patients. However, the incidence of injury is likely underestimated. Moreover, these injuries represent a significant surgical challenge for experienced surgeons [1, 23].

Surgical treatment of injured ligament structures seems to achieve better clinical outcomes than nonsurgical treatment. An injury to three or more ligaments is associated with a greater risk of post‐operative stiffness [17], and it is recommended to perform concurrent reconstruction of all injured ligaments to reduce the risk of graft failure and to promote early knee motion [6, 11, 16].

Current surgical techniques include the need to create several tunnels in a single distal femur. Due to the limited bone mass of the femur, there is a high risk of convergence between tunnels. However, the risk of tunnel collision during anatomical reconstruction can be reduced by choosing an appropriate direction.

Different angles have been described to drill tunnels on both the axial and coronal planes, as well as different tunnel depths and diameters. However, there is no consensus on the optimal surgical technique [3, 7, 8, 16, 19]. In addition, the risk of tunnel convergence may be greater in smaller knees [3, 16, 19], and it has been observed that the depth of tunnels also plays an important role [2, 8].

Patient‐specific instrumentation (PSI) is useful in performing various procedures around the knee, such as osteotomies or knee replacements, thus allowing them to be performed with greater precision [9, 18]. The need to improve the accuracy of femoral tunnel positioning remains a significant surgical challenge. In the context of multi‐ligament knee injuries, PSI has demonstrated promising results in tunnel drilling in experimental studies, improving surgical accuracy in tunnel placement as well as in terms of reproducibility [5]. The purpose of this study was to compare the accuracy of PSI and the conventional freehand technique performed by a surgeon with level 3 experience [22] in drilling lateral and medial femoral tunnels in multi‐ligament knee injuries.

## METHODS

This was a randomized experimental surgery study based on human cadaveric models. This study was performed in accordance with the relevant guidelines and regulations. All experimental protocols were approved by the Parc Taulí Clinical Research Ethics Committee with registration number 2022/3027. The cadaveric knee models were obtained from the Scientific Anatomy Center, SL (Valencia, Spain), which was responsible for providing informed consent and preconditioning the anatomical models before they were supplied. Ten cadaveric knees were used and divided into two groups: five knees were in the PSI group, and the other five knees were in the control group, which underwent conventional freehand surgery.

Of the 10 knees, four were from women (two right and two left) and six were from men (four right and two left). The mean age of the specimens was 76 years.

### Surgical planning

Surgical planning was performed on each knee. First, a preoperative computed tomography (CT) scan of each cadaveric knee was performed via a Discovery PET/CT 690 system (GE Healthcare). The images obtained presented the following characteristics: DICOM‐type files, a maximum slice thickness of 1 mm, contiguous or overlapping slices (no gaps allowed), a matrix size of 512 × 512, and an anatomical region default kernel (standard or high resolution) of 90‒120 kVp. Next, segmentation of the region of interest was carried out via Materialise Mimics Medical v25.0 software (Mimics Innovation Suite, Materialise MV). Segmentation resulted in 3D mesh files, which were exported to 3D modelling software called 3‐matic Medical 17.0 (Mimics Innovation Suite, Materialise MV). This software was used to perform surgical planning (for all cadavers) and surgical guide design (for only five cadavers). For this purpose, the anatomical femoral attachments of the LCL and popliteal tendon (PT) on the lateral side [4] and the MCL and posterior oblique ligament (POL) on the medial side [12] were identified. Then, four bone tunnels were planned for each knee starting from the anatomical attachments of the LCL, PT, MCL and POL in accordance with the directions recommended by Gelber et al. [7, 8]. PT tunnels were planned at 30° anteriorly in the axial plane and 30° proximally in the coronal plane. LCL tunnels were planned at 30° anteriorly in the axial plane and 0° in the coronal plane. MCL and POL femoral tunnels were planned at 30° anteriorly and proximally in both the axial and coronal planes.

Finally, to reproduce the femoral angulations, a coordinate system was created for each knee. This coordinate system was designed using the methods described by Miranda et al. [14]. It defines both axial and coronal planes and allows direction tunnel planning.

### Guide design

After the bone tunnels were planned, two surgical PSIs for each of the five knees within the PSI group were designed to reproduce the planned tunnels during surgery: one for the lateral side (LCL and PT reconstruction) and another for the medial side (MCL and POL reconstruction). The planned tunnel direction is achieved by creating cannulas in the chosen direction, through which Kirschner wires are inserted. The cannulas are 0.6 mm larger than the measurement of the Kirschner wires, which was 2.5 mm, to allow proper sliding through the PSI. These Kirschner wires are used as guidelines for subsequently performing drilling. To correctly position the guide without damaging the remaining ligament and capsule attachments, all PSIs were designed with 0.4 mm of tolerance between the bone and the PSI. The PSI was designed by both medical and engineering teams to account for both the anatomical and engineering aspects learned from previous studies [5]. Therefore, the PSI design was thin, with an average thickness for the lateral and medial PSIs of 3.3 mm and a standard deviation of 0.03 mm, thus enabling it to be cut because the Kirschner wires had diverging directions.

### 3D printing of surgical guides

**Figure 1:**
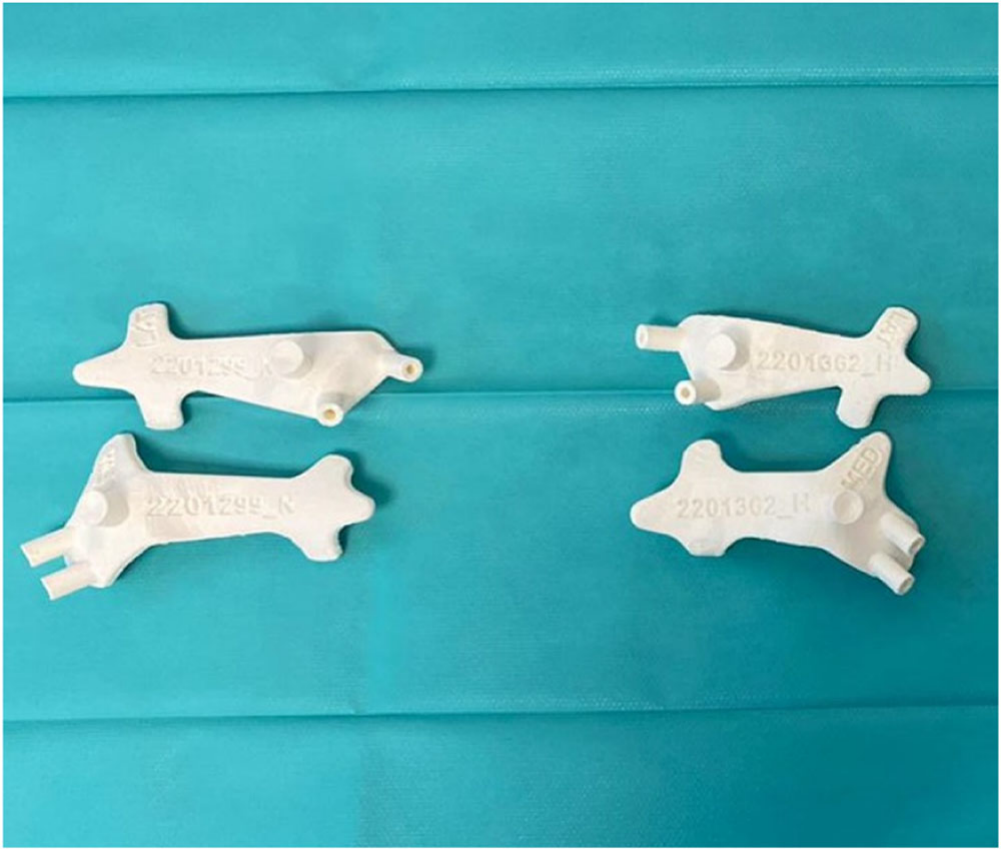
There are four PSIs for two knees. Two medial and two lateral. The two PSI at the top of the image are the lateral guides, and the ones at the bottom are the medial guides (labelled as LAT and MED). PSI, patient‐specific instrumentation.

For the five cadavers subjected to surgical guides, 10 PSIs (5 for the medial side and 5 for the lateral side) were printed with fused deposition modelling technology using polylactic acid (PLA) as the main material and polyvinyl alcohol (PVA) as the support material. Specifically, an Ultimaker S5 printer (Ultimaker) was used. The printing nozzle configuration was AA0.4 for the PLA material and BB0.4 for the PVA material. Each surgical guide was printed with a 0.1‐mm profile via Ultimaker Cura 4.0 printing software (Ultimaker) (Figure 1).

### Surgical management

#### Lateral approach

A 5‐cm lateral incision was made. The iliotibial band was subsequently opened, and the approach was distally extended between the Gerdy tubercle and the fibular head. Dissection was performed at this location in the proximal and distal directions, exposing the lateral epicondyle until the LCL and PT femoral attachments were visualized. Next, minimal subperiosteal proximal dissection was performed to allow proper adaptation of the PSI to the femoral bone surface. The PSI was performed with a shape featuring three points of support: two arms embracing the proximal femur and a concave distal shape to assist in the correct placement. Then, Kirschner wires were introduced into the guide holes. Finally, both tunnels were drilled with lengths of 25 mm and a diameter of 8 mm after removing the PSI. This was done following the technique described by Laprade et al. [13]. The same surgical approach was adopted for the freehand technique. Although the surgeon consulted the virtual surgical planning, he was free to decide the entry point and the direction of the tunnel.

#### Medial approach

**Figure 2:**
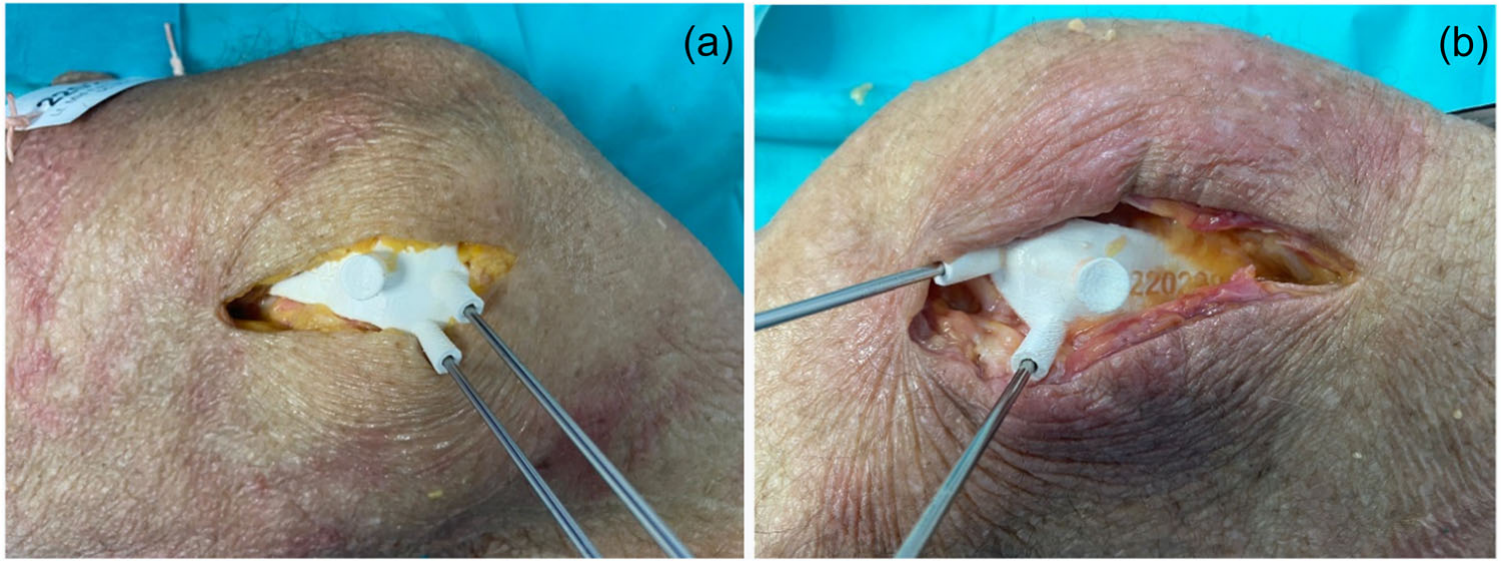
Medial (a) and lateral (b) approaches. The PSI template was correctly positioned with Kirschner wires. PSI, patient‐specific instrumentation.

An anteromedial longitudinal incision of approximately 5 cm was made over the medial epicondyle. The crural fascia was exposed, and a longitudinal incision was made down the fascia. Once the medial femoral epicondyle was exposed, the adductor magnus tendon, MCL and POL attachments were identified. Then, subperiosteal dissection was performed. Once the PSI was adapted to the bone surface, Kirschner wires were inserted to define the tunnel direction (Figure 2). After the guide was removed, the bone tunnels were drilled with a diameter of 7 mm and a depth of 25 mm, as recommended by some authors [10]. The same surgical approach was adopted for the freehand technique.

### Accuracy analysis

**Figure 3:**
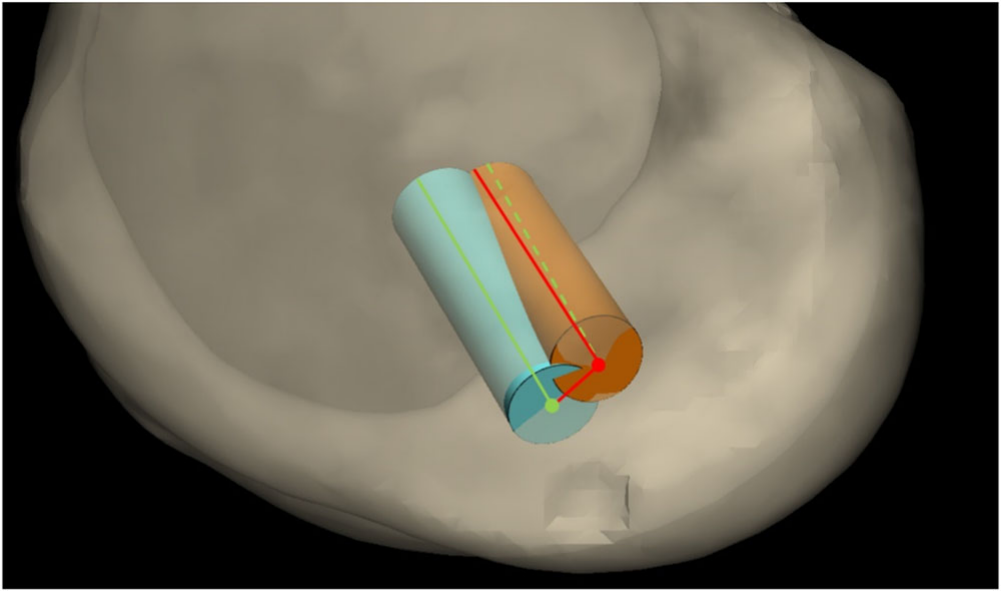
Overlay of 3D reconstructions made from pre‐ and post‐operative CT scans. Measurements of the entry point distance and angular deviation between the planned (blue) and post‐operative (orange) tunnels. CT, computerized tomography.

Post‐operative CT scans of each cadaveric knee were performed. This was followed by segmentation and the creation of 3D files such as those used in the preoperative procedure. The accuracy of the use of PSI was assessed by overlaying post‐operative and preoperative 3D bone files. The overlaying of both femurs was performed with an iterative closest point (ICP) automatic algorithm that was previously programmed by Materialise software. The distances between the entry points (measured in millimetres) of the performed and planned tunnels were analyzed. Then, the angular deviation measured in degrees was analyzed. The angular deviation was defined as the angle between two intersecting vectors. In this case, the vectors are the preoperative and post‐operative tunnels. They are calculated in the 3D plane in which the angular deviation is the greatest, as shown in Figure 3.

### Statistical analysis

For all continuous data, the median was used as the central tendency measure, and interquartile ranges (Q1–Q3) were used as the measure of variance. The Mann‒Whitney test was used to compare variables between the groups, with *p* < 0.05 considered to indicate statistical significance. A Levene test was used to compare the variance between the two methods, with *p* < 0.05 considered to indicate statistical significance.

## RESULTS

The study included 10 cadaveric knees with a mean donor age of 76 years. Of these, 40% were from women and 60% from men. Additionally, 60% of the specimens were right knees, and 40% were left knees.

**Figure 4:**
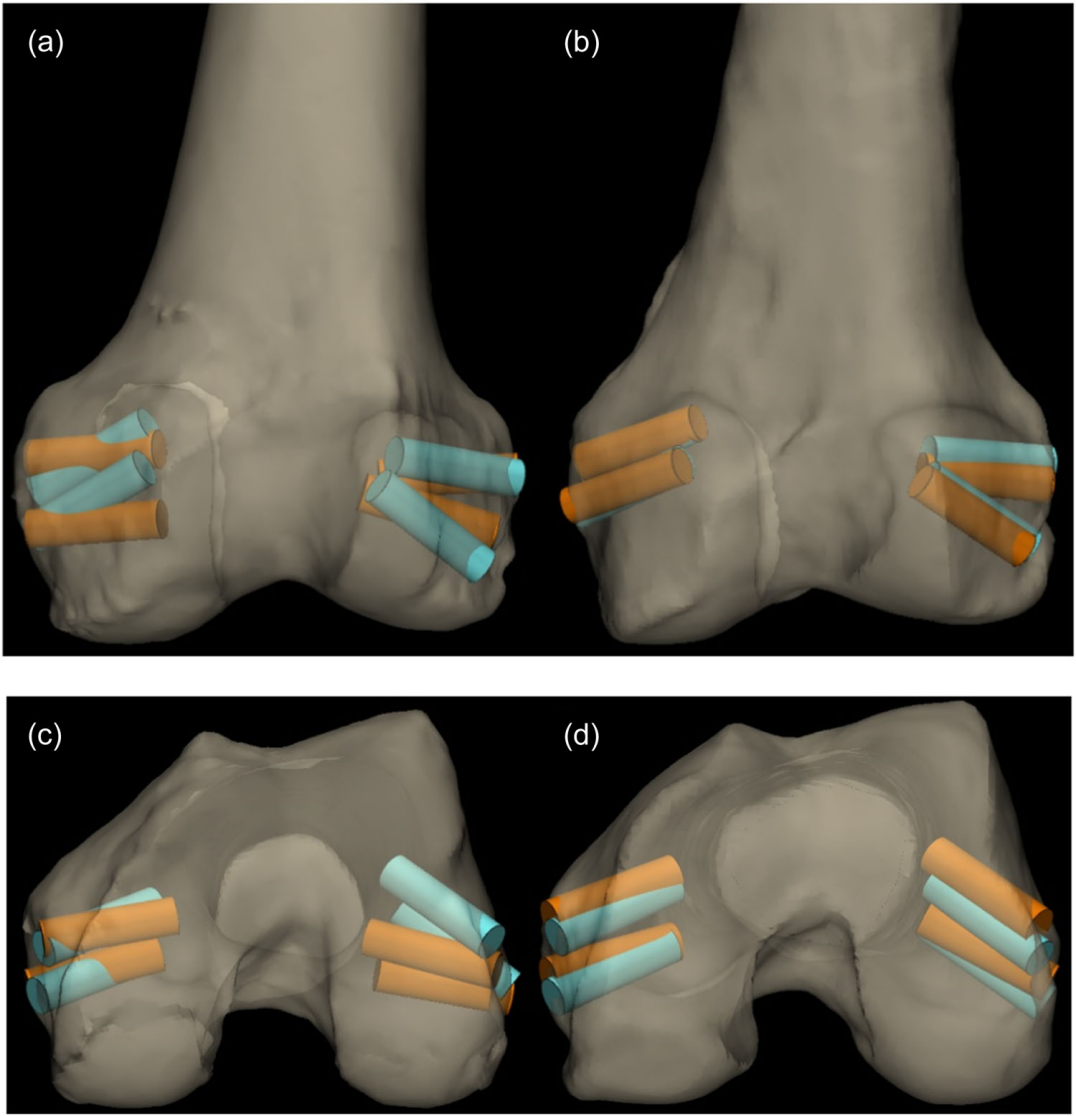
(a–d) Overlay of pre‐ and post‐operative 3D CT reconstructions. (a) Coronal view. Angular deviation between planned (blue) and post‐operative (orange) tunnelling in the control group. (b) Coronal view. Angular deviation between planned (blue) and post‐operative (orange) tunnelling in the PSI group. (c) Axial view. Angular deviation between planned (blue) and post‐operative (orange) tunnelling in the control group. (d) Axial view. Angular deviation between planned (blue) and post‐operative (orange) tunnelling in the PSI group. CT, computerized tomography; PSI, patient‐specific instrumentation.

**Figure 5:**
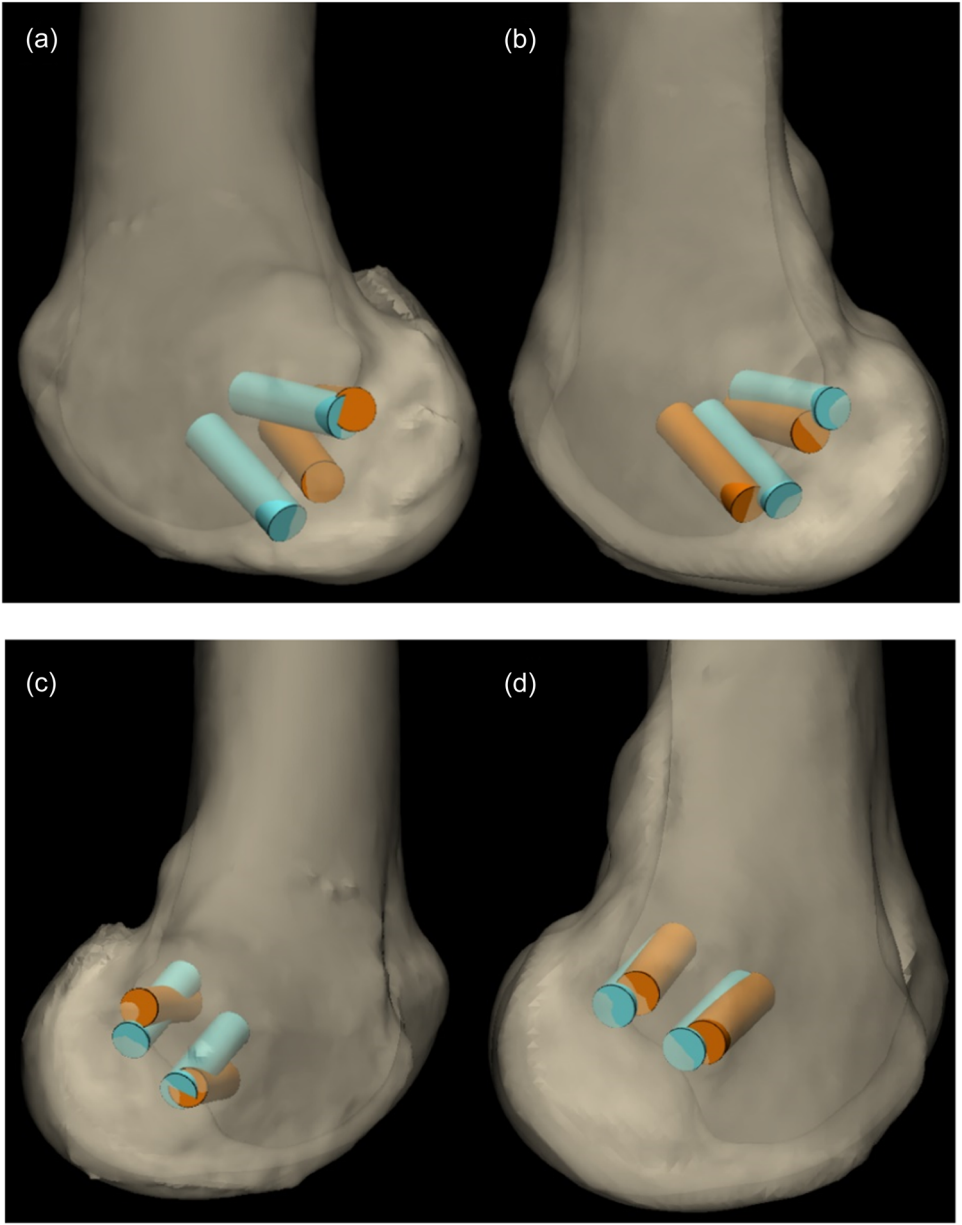
(a–d). Overlay of preoperative and post‐operative 3D CT reconstructions. (a) Lateral view. Angular deviation between planned (blue) and post‐operative (orange) tunnelling in the control group. (b) Lateral view. Angular deviation between planned (blue) and post‐operative (orange) tunnelling in the PSI group. (c) Medial view. Angular deviation between planned (blue) and post‐operative (orange) tunnelling in the control group. (d) Medial view. Angular deviation between planned (blue) and post‐operative (orange) tunnelling in the PSI group. PSI, patient‐specific instrumentation.

Table 1 shows the global angular deviation in degrees and entry point distance in mm between the planned and postsurgical tunnels. Tables 2 and 3 and Figures 4a–d and 5a‐d show the angular deviation and entry point distance between the planned and postsurgical tunnels for each ligament.

**Table 1:** Parameters for angular deviation in degrees and entry point distance in mm between the control and PSI groups for all ligaments.

Outcomes	Control (freehand), *N* = 20	PSI, *N* = 20	*p*
Angular deviation	22.3 [17.7–25.2]	5.7 [4.0–8.2]	<0.001,^a^ <0.02^b^
Entry point distance	5.5 [2.6–8.8]	4.2 [3.6–5.7]	0.369,^a^ <0.0003^b^

Abbreviation: PSI, patient‐specific instrumentation.

^a^ Mann‒Whitney *U* test.

^b^ Levene test.

**Table 2:** Parameters for angular deviation in degrees between the control and PSI groups for each ligament.

Outcomes	Control (freehand), *N* = 5	PSI, *N* = 5	*p*
LCL	17.4 [12.7–23.8]	5.7 [3.3–6.6]	0.008^a^
PT	20.7 [16.5–25.2]	4.4 [2.6–8.3]	0.008^a^
MCL	23.8 [17.8–25.8]	7.5 [5.4–8.7]	0.008^a^
POL	24.7 [21.6–27.8]	4.8 [3.2–10.3]	0.008^a^

Abbreviations: LCL, lateral collateral ligament; MCL, medial collateral ligament; POL, posterior oblique ligament; PSI, patient‐specific instrumentation; PT, popliteal tendon.

^a^ Mann‒Whitney *U* test.

**Table 3:** Parameters for the entry point distance in mm between the control and PSI groups for each ligament.

Outcomes	Control (freehand), *N* = 5	PSI, *N* = 5	*p*
LCL	2.5 [2.2–5.9]	4.4 [3.5–6.3]	0.222^a^
PT	4.9 [2.5–5.4]	5.6 [4.1–6.8]	0.310^a^
MCL	6.6 [3.5–9.0]	3.8 [3.5–5.0]	0.222^a^
POL	10.7 [7.2–11.3]	3.9 [3.3–5.1]	0.008^a^

Abbreviations: LCL, lateral collateral ligament; MCL, medial collateral ligament; POL, posterior oblique ligament; PSI, patient‐specific instrumentation; PT, popliteal tendon.

^a^ Mann–Whitney *U* test.

The average distance error achieved during the ICP algorithm for the overlay of the preoperative and post‐operative CT scans was 0.63 mm.

No significant differences were found in the distance from the entry point between the control group and the PSI group. However, there were significant differences in angular deviation in favour of the PSI group (*p* < 0.001).

## DISCUSSION

The most important finding in this study is that PSI yielded greater accuracy in terms of angular deviation than the standard freehand procedure. The difference in angular deviation between the PSI group and the control group was statistically significant (*p* < 0.001). Moreover, a difference in the IQR of angular deviation was observed, thus indicating that the PSI showed greater reliability and reproducibility.

Several studies in the literature have explored the optimal direction for tunnel drilling to prevent tunnel convergence or invasion of the trochlea. Moatshe et al. suggested that convergence with the ACL tunnel could be eliminated by orienting the LCL and PT tunnels 35° anteriorly [16]. Gelber et al. suggested that when performing posterolateral reconstructions in combination with simultaneous anterior and posterior cruciate ligament procedures, PT tunnels should be drilled at 30° anteriorly in axial plane and 30° proximally in coronal plane to avoid collision [7]. Similarly, Gelber et al. suggested that LCL tunnels should be drilled at 30° anteriorly in axial plane and 0° in coronal plane to avoid the same collision [7]. On the other hand, Kimm et al. reported that, to avoid collision between the ACL tunnel and posterolateral corner reconstruction, LCL and PT tunnelling are safe when placed at angles of approximately 20° anterior and 10° proximal to the transepicondylar axis [21]. Tunnel collision during simultaneous multiple‐ligament reconstruction also depends on anatomical factors, such as the width of the lateral femur condyle, as well as technical factors, such as the ACL reconstruction method chosen, the degree of knee flexion during ACL tunnel drilling, and the angle used to drill the LCL and PT tunnels. Other authors drilled LCL and PT tunnels at 20° in the coronal plane and 20° in the axial plane while simultaneously reconstructing the ACL. They asserted that angulation is safe for reducing the possibility of tunnel confluence [3, 19].

The results showed that in the control group, there may be a variation of approximately 20° with respect to what is planned. As there are fewer degrees of angular deviation and less variability in all measurements in the PSI group, our findings suggest that the use of this technique could lead to less intraoperative tunnel confluence. In this study, no cases of tunnel confluence or collision were observed in either the PSI group or the control group.

Regarding the medial side, Moatshe et al. reported that to avoid convergence between the MCL and PCL, the MCL tunnel should be oriented at 40° proximally and anteriorly, and the POL tunnel should be oriented at 20° proximally and anteriorly. Moatshe et al. also suggested that POL and PT tunnels oriented at 0° in the axial plane had a greater risk of violating the intercondylar notch [16]. Gelber et al. suggested that in simultaneous posteromedial reconstructions and posterior cruciate ligament procedures, the MCL and POL femoral tunnels should be drilled at 30° anteriorly and proximally in both the axial and the coronal planes. The POL femoral tunnel can also be angled at 0° in the coronal plane, although it is shorter from the intercondylar notch and has a greater risk of collision with PCL tunnels [8].

During ACL reconstruction, lateral augmentation techniques may be used to reduce rotational instability. When reconstructing the anterolateral ligament, it is crucial to adjust the tunnel direction to prevent convergence with the ACL tunnel [15]. As noted in previous studies, the ACL reconstruction tunnel should be oriented posteriorly (with an alpha angle >40°) to minimize the risk of tunnel collision or trochlear damage. The tunnel orientation should follow one of these three configurations: an axial angle of 40° anteriorly and a coronal angle of 10° proximally, an axial angle of 35° anteriorly and a coronal angle of 5° proximally or an axial angle of 30° anteriorly and a coronal angle of 0°. These orientations have been associated with a low risk of tunnel collision and trochlear damage [20].

The second finding in this study was that the difference in distance from the entry point between the PSI group and the control group was not statistically significant. One explanation for this result may be that the surgeon determined the entry point of each tunnel, visualizing the anatomical footprints of each ligament to perform the tunnel at the correct entry point. This might explain the minimal differences found between the two groups. However, when the variance was evaluated with the Levene test, the results were statistically significant (*p* < 0.0003), indicating fewer outliers and more precision in the PSI group.

When interpreting these results, it is important to consider the limitations of the study. Neither the relationship between posterolateral complex reconstruction, anterior cruciate tunnel reconstruction and anterolateral tunnel reconstruction nor the relationship between the posteromedial complex and posterior cruciate reconstruction was evaluated. Although the use of PSI is a precise tool, it cannot be asserted that there is a reduced risk of tunnel convergence in the reconstruction of all ligaments. Further research should be conducted to study whether the concurrent reconstruction of the ACL, PCL, MCL (and POL) and LCL (and PT) with PSI can be more accurate than freehand reconstruction and lead to less tunnel convergence.

In addition, this was a cadaveric study. Although a minimally invasive approach was used, it is not yet known whether distal femoral soft tissue detachment to correctly adapt PSI would be different in a patient. It would be worthwhile to evaluate this point in a clinical trial.

## CONCLUSIONS

Compared with the freehand technique performed by an experienced surgeon, PSI demonstrated significantly greater accuracy in terms of the mean angular deviation.

## AUTHOR CONTRIBUTIONS

Núria Fernández‐Poch contributed to the conception and design of the study, experimental surgery, and interpretation of data and drafted the manuscript. Ferran Fillat‐Gomà contributed to the conception and design of the study, virtual surgical planning and surgical guides creation, experimental surgery and interpretation of data. Giovanni Grillo contributed to the conception, design and coordination of the study, data analysis and interpretation of data. Mireia Gamundi performed virtual surgical planning and surgical guide creation. Christian Yela‐Verdú contributed to the conception and performance of the experimental surgeries. Sergi Gil‐Gonzalez contributed to the conception and performance of the experimental surgeries. Xavier Pelfort contributed to the conception and design of the study, virtual surgical planning, experimental surgery and interpretation of data and helped to draft the manuscript. The authors read and approved the final manuscript.

## CONFLICT OF INTEREST STATEMENT

Ferran Fillat‐Gomà holds stocks of Tailor Surgery SL. Ferran Fillat‐Gomà has another affiliation organization not related to this study but related to orthopaedic surgery, Department of Orthopedic and Trauma Surgery, Hospital Clinic Barcelona, University of Barcelona. The remaining authors declare no conflicts of interest.

## ETHICS STATEMENT

The approval from Parc Taulí's Clinical Research and Ethics Committee was obtained, with registration number 2022/3027. This work is an experimental study using purchased cadaveric knees.

## Data Availability

All data generated or analyzed during this study are included in this published article.

## References

[1] Alentorn‐Geli E , Lazarides AL , Utturkar GM , Myers HS , Samuelsson K , Choi JHJ , et al. Factors predictive of poorer outcomes in the surgical repair of multiligament knee injuries. Knee Surg Sports Traumatol Arthrosc. 2019;27:445–459. 30083969 10.1007/s00167-018-5053-9

[2] Camarda L , Grassedonio E , Lauria M , Midiri M , D'Arienzo M . How to avoid collision between PCL and MCL femoral tunnels during a simultaneous reconstruction. Knee Surg Sports Traumatol Arthrosc. 2016;24:2767–2772. 25429764 10.1007/s00167-014-3446-y

[3] Gali JC , Bernardes AP , dos Santos LC , Ferreira TC , Almagro MAP , da Silva PAC . Tunnel collision during simultaneous anterior cruciate ligament and posterolateral corner reconstruction. Knee Surg Sports Traumatol Arthrosc. 2016;24:195–200. 25288339 10.1007/s00167-014-3363-0

[4] Chahla J , Moatshe G , Dean CS , Laprade RF . Posterolateral corner of the knee: current concepts. Arch Bone Jt Surg. 2016;4:97–103. 27200384 PMC4852053

[5] Fernández‐Poch N , Fillat‐Gomà F , Martínez‐Carreres L , Coderch‐Navarro S , Yela‐Verdú C , Carbó‐Cedán S , et al. Three‐dimensional‐printed patient‐specific instrumentation is an accurate tool to reproduce femoral bone tunnels in multiple‐ligament knee injuries. Int Orthop. 2023;47:1213–1219. 36799973 10.1007/s00264-023-05712-1 PMC10079717

[6] G.Geeslin A , LaPrade RF . Outcomes of treatment of acute Grade‐III isolated and combined posterolateral knee injuries. J Bone Joint Surg. 2011;93:1672–1683. 21938371 10.2106/JBJS.J.01639

[7] Gelber PE , Erquicia JI , Sosa G , Ferrer G , Abat F , Rodriguez‐Baeza A , et al. Femoral tunnel drilling angles for the posterolateral corner in multiligamentary knee reconstructions: computed tomography evaluation in a cadaveric model. Arthroscopy. 2013;29:257–265. 23265690 10.1016/j.arthro.2012.08.015

[8] Gelber PE , Masferrer‐pino À , Erquicia JI , Abat F , Pelfort X , Rodriguez‐Baeza A , et al. Femoral tunnel drilling angles for posteromedial corner reconstructions of the knee. Arthroscopy. 2015;31:1764–1771. 25911395 10.1016/j.arthro.2015.03.007

[9] Jones GG , Jaere M , Clarke S , Cobb J . 3D printing and high tibial osteotomy. EFORT Open Rev. 2018;3:254–259. 29951264 10.1302/2058-5241.3.170075 PMC5994616

[10] Laprade RF , Wijdicks CA . Surgical technique. Development of an anatomic medial knee reconstruction. Clin Orthop Relat Res. 2012;470:806–814. 21909850 10.1007/s11999-011-2061-1 PMC3270176

[11] Laprade RF , Chahla J , Dephillipo NN , Cram T , Kennedy MI , Cinque M , et al. Single‐stage multiple‐ligament knee reconstructions for sports‐related injuries outcomes in 194 patients. Am J Sports Med. 2019;47:2563–2571. 31381372 10.1177/0363546519864539

[12] Laprade RF , Engebretsen AH , Ly TV , Johansen S , Wentorf FA , Engebretsen L . The anatomy of the medial part of the knee. J Bone Joint Surg Am. 2007;89:2000–2010. 17768198 10.2106/JBJS.F.01176

[13] LaPrade RF , Johansen S , Wentorf FA , Engebretsen L , Esterberg JL , Tso A . An analysis of an anatomical posterolateral knee reconstruction: an in vitro, biomechanical study and development of a surgical technique. Am J Sports Med. 2004;32:1405–1414. 15310564 10.1177/0363546503262687

[14] Miranda DL , Rainbow MJ , Leventhal EL , Crisco JJ , Fleming BC . Automatic determination of anatomical coordinate systems for three‐dimensional bone models of the isolated human knee. J Biomech. 2010;43:1623–1626. 20167324 10.1016/j.jbiomech.2010.01.036 PMC2866785

[15] Mitrousias V , Chalatsis G , Komnos G , Neri T , Hantes M . Lateral augmentation procedures in anatomic anterior cruciate ligament reconstruction. How to avoid tunnel collision with intraoperative tunnel visualization: a technical note. J ISAKOS. 2023;8:137–139. 36921765 10.1016/j.jisako.2023.03.001

[16] Moatshe G , Brady AW , Slette EL , Chahla J , Turnbull TL , Engebretsen L , et al. Multiple ligament reconstruction femoral tunnels: intertunnel relationships and guidelines to avoid convergence. Am J Sports Med. 2017;45:563–569. 27872126 10.1177/0363546516673616

[17] Özbek EA , Dadoo S , Grandberg C , Runer A , Cong T , Hughes JD , et al. Early surgery and number of injured ligaments are associated with postoperative stiffness following multi‐ligament knee injury surgery: a systematic review and meta‐analysis. Knee Surg Sports Traumatol Arthrosc. 2023;31:4448–4457. 37486368 10.1007/s00167-023-07514-9

[18] Qiu B , Liu BSF , Tang B , et al Clinical study of 3D imaging and 3D printing technique for patient‐specific instrumentation in total knee arthroplasty. J Knee Surg. 2017;822–828. 10.1055/s-0036-1597980 28122388

[19] Shuler MS , Jasper LE , Rauh PB , Mulligan ME , Moorman CT . Tunnel convergence in combined anterior cruciate ligament and posterolateral corner reconstruction. Arthroscopy. 2006;22:193–198. 16458805 10.1016/j.arthro.2005.12.001

[20] Stordeur A , Grange S , Servien E , Blache Y , Klasan A , Putnis SE , et al. Optimal combination of femoral tunnel orientation in anterior cruciate ligament reconstruction using an inside‐out femoral technique combined with an anterolateral extra‐articular reconstruction. Am J Sports Med. 2022;50:1205–1214. 35244477 10.1177/03635465221078326

[21] Kim SJ , Chang CB , Choi CH , Yoo YS , Kim SH , Ko JH , et al. Intertunnel relationships in combined anterior cruciate ligament and posterolateral corner reconstruction: an in vivo 3‐dimensional anatomic study. Am J Sports Med. 2013;41:849–857. 23467553 10.1177/0363546513478571

[22] Tang JB , Giddins G . Why and how to report surgeons' levels of expertise. J Hand Surg Eur Vol. 2016;41(4):365–366. 27076602 10.1177/1753193416641590

[23] Ng JWG , Myint Y , Ali FM . Management of multiligament knee injuries. EFORT Open Rev. 2020;5:145–155. 32296548 10.1302/2058-5241.5.190012 PMC7144894

